# Surfactant Protein-D Is Essential for Immunity to Helminth Infection

**DOI:** 10.1371/journal.ppat.1005461

**Published:** 2016-02-22

**Authors:** Sumaiyya Thawer, Jennifer Auret, Corinna Schnoeller, Alisha Chetty, Katherine Smith, Matthew Darby, Luke Roberts, Rosie-Marie Mackay, Harry J. Whitwell, John F. Timms, Jens Madsen, Murray E. Selkirk, Frank Brombacher, Howard William Clark, William G. C. Horsnell

**Affiliations:** 1 Institute of Infectious Disease and Molecular Medicine, International Centre for Genetic Engineering and Biotechnology and Division of Immunology, University of Cape Town, Cape Town, South Africa; 2 Department of Life Sciences, Imperial College London, London, United Kingdom; 3 Institute of Infection and Immunity, University of Cardiff, Cardiff, United Kingdom; 4 Clinical & Experimental Sciences Academic Unit, Southampton General Hospital, University of Southampton, Southampton, United Kingdom; 5 Cancer Proteomics, Institute for Women’s Health, University College London, London, United Kingdom; University of Manchester, UNITED KINGDOM

## Abstract

Pulmonary epithelial cell responses can enhance type 2 immunity and contribute to control of nematode infections. An important epithelial product is the collectin Surfactant Protein D (SP-D). We found that SP-D concentrations increased in the lung following *Nippostrongylus brasiliensis* infection; this increase was dependent on key components of the type 2 immune response. We carried out loss and gain of function studies of SP-D to establish if SP-D was required for optimal immunity to the parasite. *N*. *brasiliensis* infection of SP-D-/- mice resulted in profound impairment of host innate immunity and ability to resolve infection. Raising pulmonary SP-D levels prior to infection enhanced parasite expulsion and type 2 immune responses, including increased numbers of IL-13 producing type 2 innate lymphoid cells (ILC2), elevated expression of markers of alternative activation by alveolar macrophages (alvM) and increased production of the type 2 cytokines IL-4 and IL-13. Adoptive transfer of alvM from SP-D-treated parasite infected mice into naïve recipients enhanced immunity to *N*. *brasiliensis*. Protection was associated with selective binding by the SP-D carbohydrate recognition domain (CRD) to L4 parasites to enhance their killing by alvM. These findings are the first demonstration that the collectin SP-D is an essential component of host innate immunity to helminths.

## Author Summary

Infections by parasitic worms are very common, and controlling them is a major medical and veterinary challenge. Very few drugs exist to treat them, and the parasites can develop resistance to these. In order to find new ways to control worm infections, understanding how our immune system responds to them is essential. Many important parasitic worm infections move through the host lung. In this study we show that a major secreted protein in the lung, Surfactant Protein D (SP-D), is essential for immunity to a parasitic worm infection. We found that this protein binds to worm larvae in the lung to help the immune system kill them. Infecting mice that do not express SP-D with worms demonstrates SP-D is important in this immune response. These mice are unable to launch an effective anti-worm immune response and have many more worms in their intestine compared to mice that do express SP-D. We also show that if we increase SP-D levels in the lung the mouse has better immunity to worms. Together this shows for the first time that SP-D is very important for immunity to worm infections.

## Introduction

Surfactant Protein (SP)-D is a constitutively expressed C-type lectin, which has a well recognized role in innate pulmonary immunity against viruses, bacteria and fungi, as well as in maintaining pulmonary homeostasis [1]. Direct SP-D interactions with immune (such as alveolar macrophages [2]) and non-immune cells [3] can protect against immune pathologies such as chronic obstructive pulmonary disorder (COPD): SP-D-deficient (SP-D-/-) mice develop spontaneous chronic lung inflammation and emphysema [4], which can be prevented by recombinant SP-D replacement [5]. SP-D binding of pathogens and allergens is also important for preventing or reducing the onset of pathology following infections such as respiratory syncytial virus (RSV) and influenza, and also protects against airway inflammation [6, 7].

SP-D is primarily secreted by alveolar epithelial type II (ATII) cells [1]; ATII cells also secrete type 2 associated alarmins such as IL-33, which are important for immunity to helminth infections [8]. SP-D also both controls and is controlled by type 2 immune responses; the canonical type 2 cytokines IL-4 and IL-13 enhance pulmonary SP-D concentrations, yet in the absence of SP-D CD4+ TH2 cytokine levels are raised [9]. SP-D therefore is likely to play an important role in limiting overzealous type 2 responses and immune-associated pathology in the lung. Control of TH2 associated immune pathologies can also be achieved by induction of regulatory innate immune cell phenotypes, such as alternatively activated macrophages (AAM) [10]. SP-D can also interact directly with myeloid cells to enhance antigen or pathogen clearance by macrophages, and also to regulate potentially damaging macrophage-driven inflammatory responses [5, 11].

Helminth infections are likely to have contributed to the evolution of both type 2/TH2 immunity and associated mechanisms that regulate the strength of this response [12, 13]. Host protective immunity against helminths is typically TH2-dependent and is initiated by parasite interaction with epithelial cells, including ATII cells [8]. Regulation of the magnitude of this TH2-mediated immune response by, for example, regulatory T cells is important for preventing immunopathology [14, 15]. How innate host factors, such as C-type lectins, are induced by helminth infection to control infection or regulate host immunity is not well understood. Some C-type lectins have been associated with helminth infection and host immunity to them. For example, Dectin-2 contributes to *S*. *mansoni* driven inflammasome activation [16]. Only one previous report has identified any interaction between SP-D and helminths; specifically that SP-D binds to fucose residues on the tegument of *Schistosoma mansoni* [17] however, this study did not address if this interaction contributed to host immunity.

In the study presented here we demonstrate that infection with the experimental model nematode *Nippostrongylus brasiliensis* induced a striking type 2-dependent increase in the levels of host SP-D. This induction of SP-D was associated with an increase in type-2 anti-parasite immune responses. Moreover, we found that immunity to infection required direct interaction of SP-D with both the fourth stage (L4) larvae and host alveolar macrophages, driving the latter to an enhanced AAM phenotype. SP-D therefore represents a previously un-described but pivotal mechanistic contributor to host immunity to helminth infection.

## Results

### Increased SP-D levels following *N*. *brasiliensis* infection are dependent on IL-4/IL-13 cytokine levels and IL-4Rα expression

**Fig 1:**
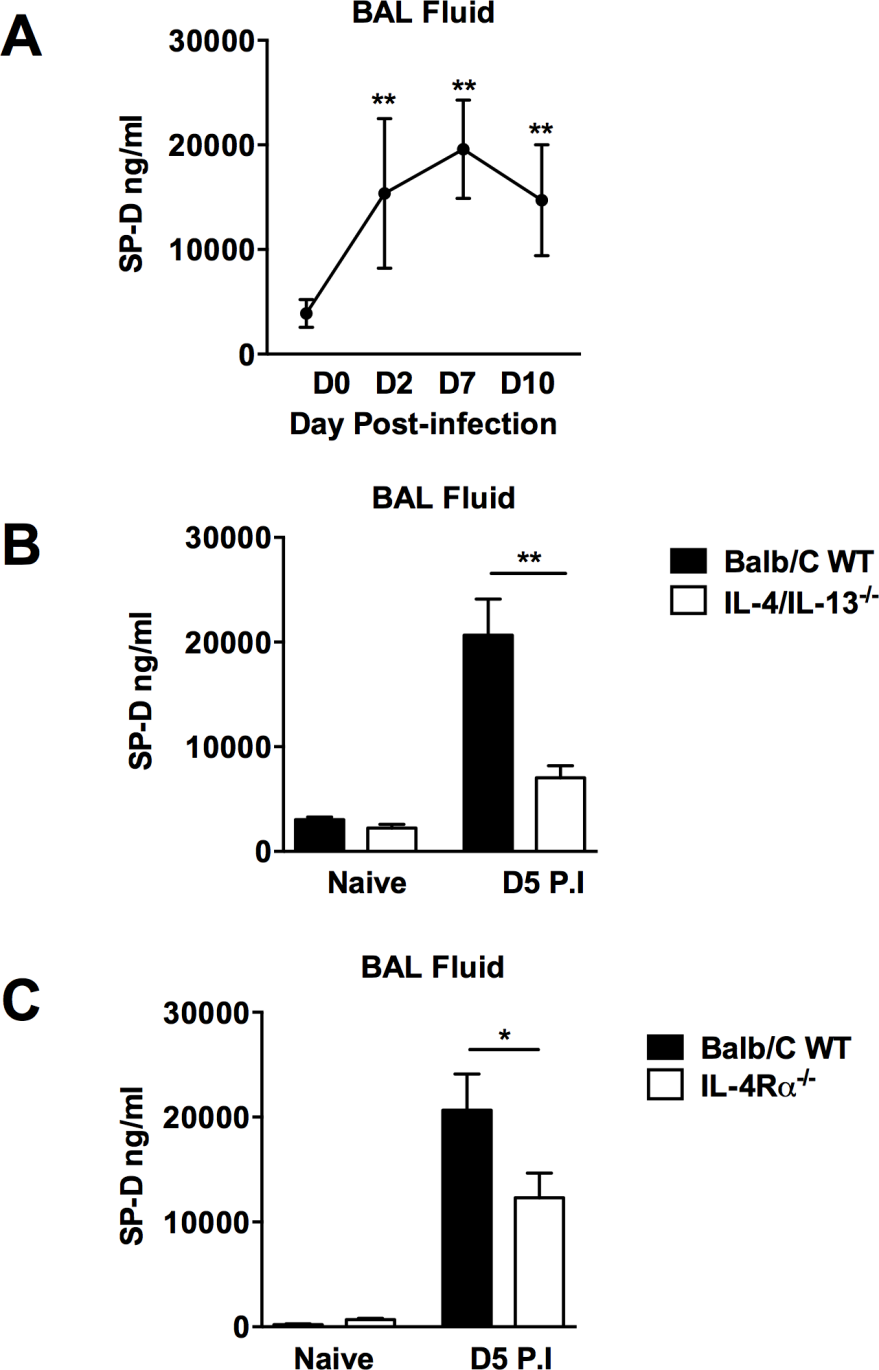
Elevation of SP-D in BAL fluid following *N*. *brasiliensis* infection is dependent on IL-4/IL-13 and IL-4Rα. SP-D levels were measured by ELISA in BAL fluid at various time points post *N*. *brasiliensis* infection (**a**). SP-D levels in IL-4/IL-13^-/-^ (**b**) and IL-4R**α** ^-/-^ mice (**c**) were measured in BAL fluid at day 5 PI and compared to wild type controls. Data are representative of 3 individual experiments. N = 5 mice per group. *P<0.05, **P<0.01.

Type 2 cytokine-associated increases in SP-D levels have previously been shown in bronchoalveolar lavage (BAL) and serum of mice following challenge with a range of antigens and pathogens [1], but not helminths. Since the lung is an important site for immunity to *N*. *brasiliensis* infection [18, 19], we examined if host immunity to *N*. *brasiliensis* infection increased pulmonary and systemic levels of SP-D. Analysis of BAL (**Fig 1A**) and serum (**S1A Fig**) of *N*. *brasiliensis*-infected mice showed a temporal relationship between SP-D levels and progression and resolution of *N*. *brasiliensis* infection. The highest levels of SP-D were found at the peak of infection; namely day 7 post primary infection in both BAL and serum, highlighting an association with host protective immunity to *N*. *brasiliensis*. SP-D production has been shown to be dependent on IL-4, IL-13 and STAT6 [9]. Immunity to *N*. *brasiliensis* results in enhanced host secretion of IL-4 and IL-13, with IL-13 being essential for resolution of infection [20]. We investigated the requirements of IL-4 and IL-13 for SP-D production in response to *N*. *brasiliensis* infection. WT, IL-4/IL-13^-/-^ mice (**Fig 1B**) and IL-4Rα^-/-^ mice (**Fig 1C**) were infected with *N*. *brasiliensis* and, at 5 days post-infection, SP-D levels in BAL fluid and serum (**S1B Fig**) were quantified. Significantly higher SP-D levels were found in WT mice when compared to both IL-4/IL-13^-/-^ and IL-4Rα^-/-^ mice.

### SP-D-/- mice demonstrate highly impaired immunity to *N*. *brasiliensis*

**Fig 2:**
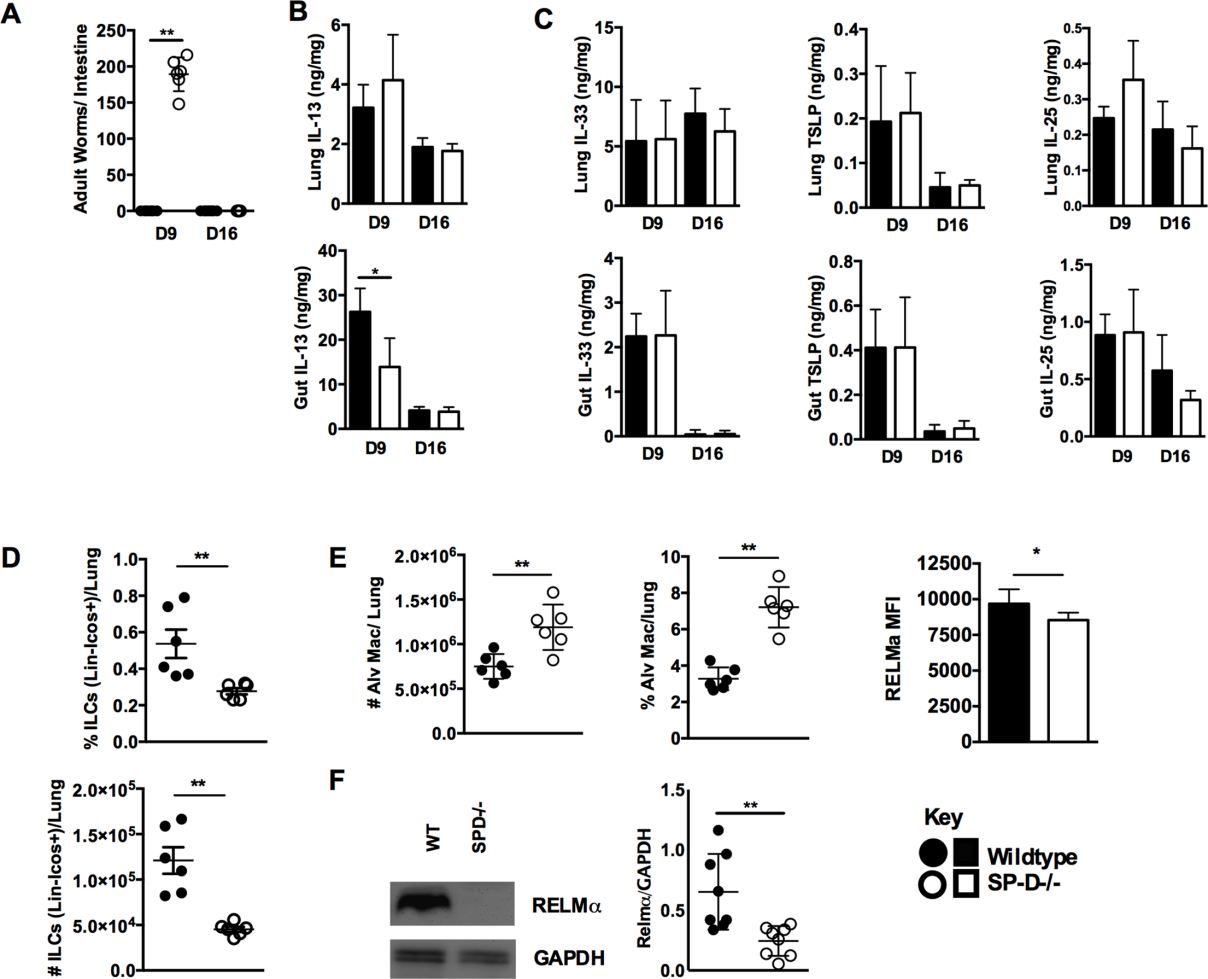
Immunity to *N*. *brasiliensis* is impaired in SP-D-/- mice. *N*. *brasiliensis* infected wild type and SP-D-/- mice were killed at days 9 and 16 PI. Intestinal worm burdens were quantified **(a)**. Levels of lung and intestinal tissue IL-13 **(b)**, IL-33, TSLP and IL-25 **(c)** were established by ELISA at days 9 and 16 PI. FACS analysis established proportions and total numbers of ILC **(d)**. Numbers and proportions of alveolar macrophage and mean fluorescence intensity (MFI) of RELM-α detection within this population **(e).** Relative concentration of RELM-α in the lungs of day 9PI WT and SP-D-/- mice was detected by Western Blot and quantified by densitometry **(f).** Data are representative of 2–3 individual experiments with N = 4–6 mice per group. *P<0.05, **P<0.01.

To test the association between elevated SP-D levels and immunity to *N*. *brasiliensis* we infected wild type C57/BL-6 and SP-D-deficient (SP-D-/-) mice [21] with the parasite and examined mice at days 9 and 16 post-infection (PI). At day 9 PI SP-D-/- mice had high worm burdens while wild type mice had resolved the infection, and by day 16 PI SP-D-/- mice had resolved the infection **(Fig 2A)**. Levels of the cytokine IL-13, essential for resolution of *N*. *brasiliensis* infection, were significantly reduced at day 9 PI in the intestine, but not the lung, of SP-D-/- mice when compared to wild type mice **(Fig 2B)**. Equivalent levels of the alarmins IL-25, IL-33 and TSLP were detected in the lung and intestine of both WT and SP-D-/- mice at days 9 and 16 PI **(Fig 2C)**.

We also quantified the numbers and proportions of innate lymphoid cells (ILCs) and alveolar macrophages at day 9 post infection to identify if SP-D was required for the development of these cells, which are essential for optimal resolution of *N*. *brasiliensis* infection. Numbers and proportions of ILCs, which are required for resolution of *N*. *brasiliensis* infection [22], were significantly reduced at day 9 PI **(Fig 2D)**. Proportions and numbers of alveolar macrophages (AlvM) was significantly increased in SP-D-/- mice when compared to wild type mice **(Fig 2E)**. However, expression of a hallmark of alternative activation, resistin-like molecule (RELM) alpha/FIZZ1 (RELM-α), within AlvM was significantly decreased in SP-D-/- mice compared to wild-type by day 9 post-infection **(Fig 2E)**. Additionally, analysis of total RELM-α protein levels in the lung revealed a significant reduction of RELM-α levels in SP-D-/- mice when compared to WT, although total levels of YM1 were equivalent in both groups **(Fig 2F** and **S2 Fig)**. To establish if expression of SP-D in the lung alone was sufficient to confer protection we infected mice capable of doxycycline-inducible expression of SP-D only in the lung (CCSP-rtTA, (tetO)_7_-rSP-D,SP-D^−/−^). Significantly reduced *N*. *brasiliensis* numbers in the intestine in doxycycline treated mice (SP-D^on^) when compared to untreated mice (SP-D^off^) demonstrated that SP-D expression in the lung is a major component of host immunity against *N*. *brasiliensis* **(S2 Fig)**. However, numbers of worms in SP-D^on^ mice were significantly higher than in WT mice indicating possible SP-D mediated inputs to immunity at other sites.

### SP-D preferentially binds to lung stage L4 *N*. *brasiliensis* to enhance host immunity to the parasite

**Fig 3:**
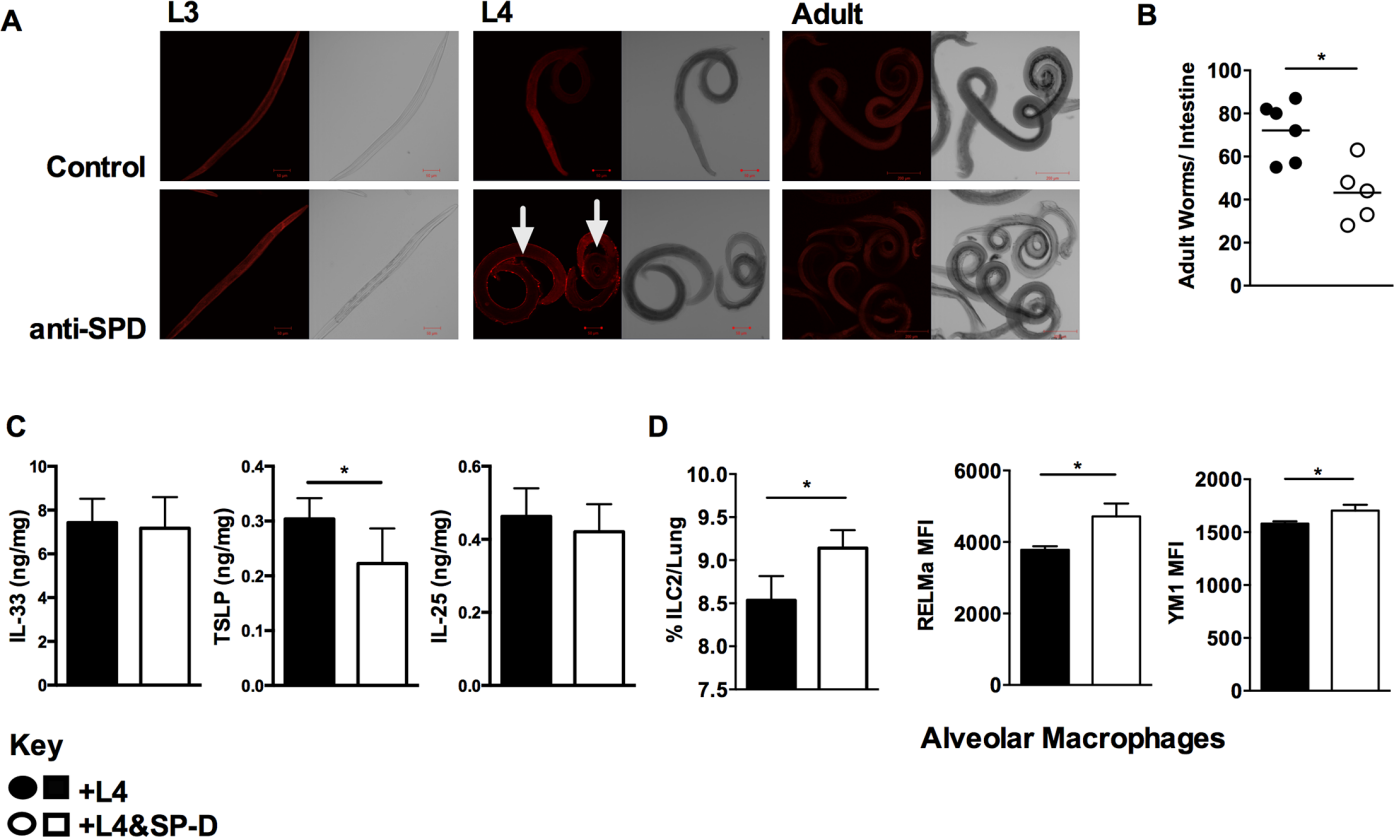
Selective SP-D binding to L4 *Nippostrongylus brasiliensis* enhances host protective immunity. Confocal microscopic images of L3, L4 and Adult stage of *N*. *brasiliensis* incubated with 20 μg/ml of rfhSP-D, biotin-conjugated anti-rfhSP-D, followed by streptavidin-cy3 **(a)**. 250 L4 stage larvae, coated or uncoated with rfhSP-D were intra-nasally administered into naïve mice. Intestinal worm burden **(b)**, detection by ELISA of IL-33, TSLP and IL-25 in the lung **(c)**, percentage of lung ILC2 and mean fluorescence intensity (MFI) of markers of alternative macrophage activation were quantified at day 5 PI. **(d)**. Data are representative of 4 individual experiments. N = 5–8 mice per group. *P<0.05, **P<0.01.

SP-D control of infection is associated with its ability to opsonize and enhance immune recognition of pathogens. We assessed the ability of a recombinant homotrimeric fragment of human SP-D (rfhSP-D) to directly bind to *N*. *brasiliensis* by immunofluorescence using third stage larvae (L3), fourth stage larvae (L4), and adult worms. rfhSP-D binding to the surface of parasites was only seen for L4 (**Fig 3A** and **S3 Fig**). Moulting of *N*. *brasiliensis* L3 to L4 takes place in the lungs of the host. This would suggest that elevated pulmonary SP-D levels seen in the lung **(Fig 1)** could induce protective immunity by coating L4 parasites and enhanced immune recognition of these parasites both in the lung and intestine. We tested this by infecting mice intranasally with rfhSP-D-coated L4, prior coating with rfhSP-D did not result in reduced parasite viability (**S3 Fig)**. Intranasal infection of mice with rfhSP-D-coated L4 resulted in reduced worm burdens when compared to mice that received L4 alone (**Fig 3B**). Examination of host alarmin responses revealed no effect on IL-33 or IL-25, but a significant reduction in TSLP **(Fig 3C)**. Recipients of rfhSP-D-coated L4 had increased numbers of ILC2 while AlvM populations showed a heightened expression of the markers of alternative activation RELM-α chitinase-3-like protein Chil3 (Ym1) (**Fig 3D**).

### Intra-nasal administration of rfhSP-D enhances protective immunity to *N*. *brasiliensis*

**Fig 4:**
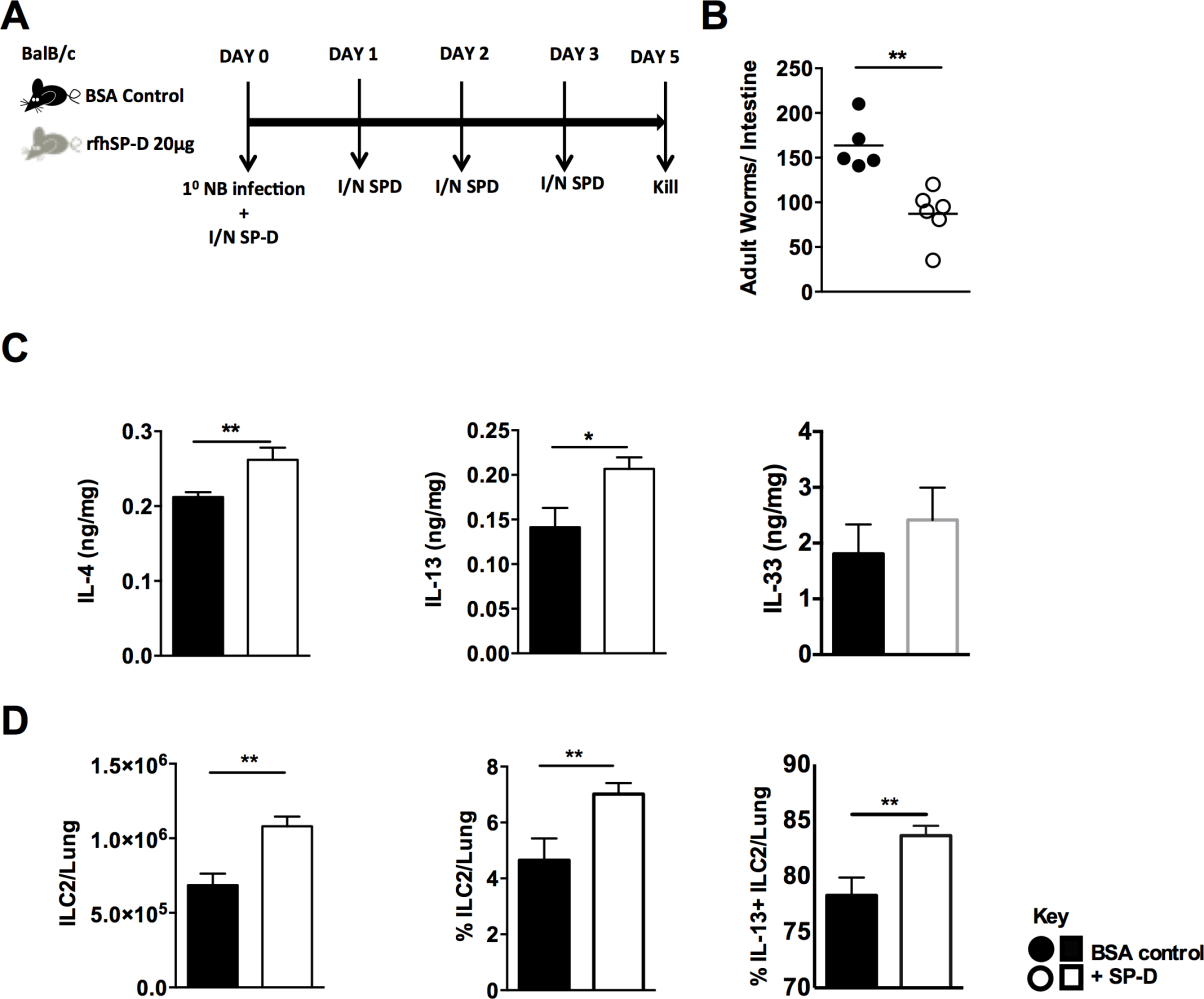
Intra-nasal administration of SP-D enhances protective immunity to *N*. *brasiliensis*. rfhSP-D treated or untreated mice infected with *N*. *brasiliensis* were killed at day 5 PI. (**a**). Intestinal worm burdens were quantified at day 5 PI. (**b**). IL-4, IL-13 and IL-33 cytokine levels in lung homogenates were detected by ELISA at day 5 PI. (**c**). Total numbers, total percentage and percentage of IL-13 positive lung ILC2s **(d)** were quantified by FACS analysis at D5 PI Black bars: control mice. White bars: SP-D treated mice. Data are representative of 2–3 experiments. N = 5–6 mice per group. *P<0.05, **P<0.01.

Having established in a loss-of-function system that SP-D is required for immunity to *N*. *brasiliensis* and that rfhSP-D binding of L4 parasites could enhance host immunity to the infection we next tested if heightened levels of pulmonary SP-D prior to *N*. *brasiliensis* infection conferred enhanced immunity against the parasite. Naïve mice treated with rfhSP-D for 4 days did not display detectable levels of IL-13 or IL4 in the lung. Numbers and proportions of ILC2s were also equivalent **(S4 Fig)**. At day 5 post-infection, intra-nasal administration of rfhSP-D prior to *N*. *brasiliensis* infection (**Fig 4A**) significantly reduced intestinal burdens of adult *N*. *brasiliensis* in rfhSP-D-treated mice compared to bovine serum albumin (BSA)-treated controls (**Fig 4B**). Protection was associated with increased pulmonary type 2 cytokines IL-4 and IL-13 but not IL-33 at day 5 post infection (**Fig 4C**). Additionally, type 2 innate lymphoid cells (ILC2) required to control *N*. *brasiliesis* infection were increased in numbers and proportions in the lung of rfhSP-D treated mice. Moreover, a higher percentage of ILC2s produced IL-13 in rfhSP-D treated mice when compared to untreated mice (**Fig 4D**).

### SP-D-treated AlvMs show increased alternative activation and enhance host immunity to *N*. *brasiliensis*

SP-D is known to regulate alveolar macrophage function [23, 24]. SP-D^-/-^ mice have a loss of homeostatic regulation of macrophage function [25, 26] which can be rescued by treatment with recombinant rat SP-D [11]. Host control of *N*. *brasiliensis* recall infection is dependent on IL-4Rα-dependent macrophage polarization to the alternatively activated phenotype in the lung [27]. AAMs are also key effector cells for controlling other helminth infections including *Heligmosomoides polygyrus* [28] and *Schistosoma mansoni* [29]. As SP-D enhanced immunity to *N*. *brasiliensis* in the lung, we hypothesized that this may be due, at least in part, to an effect on polarisation of AlvM to AAM.

**Fig 5:**
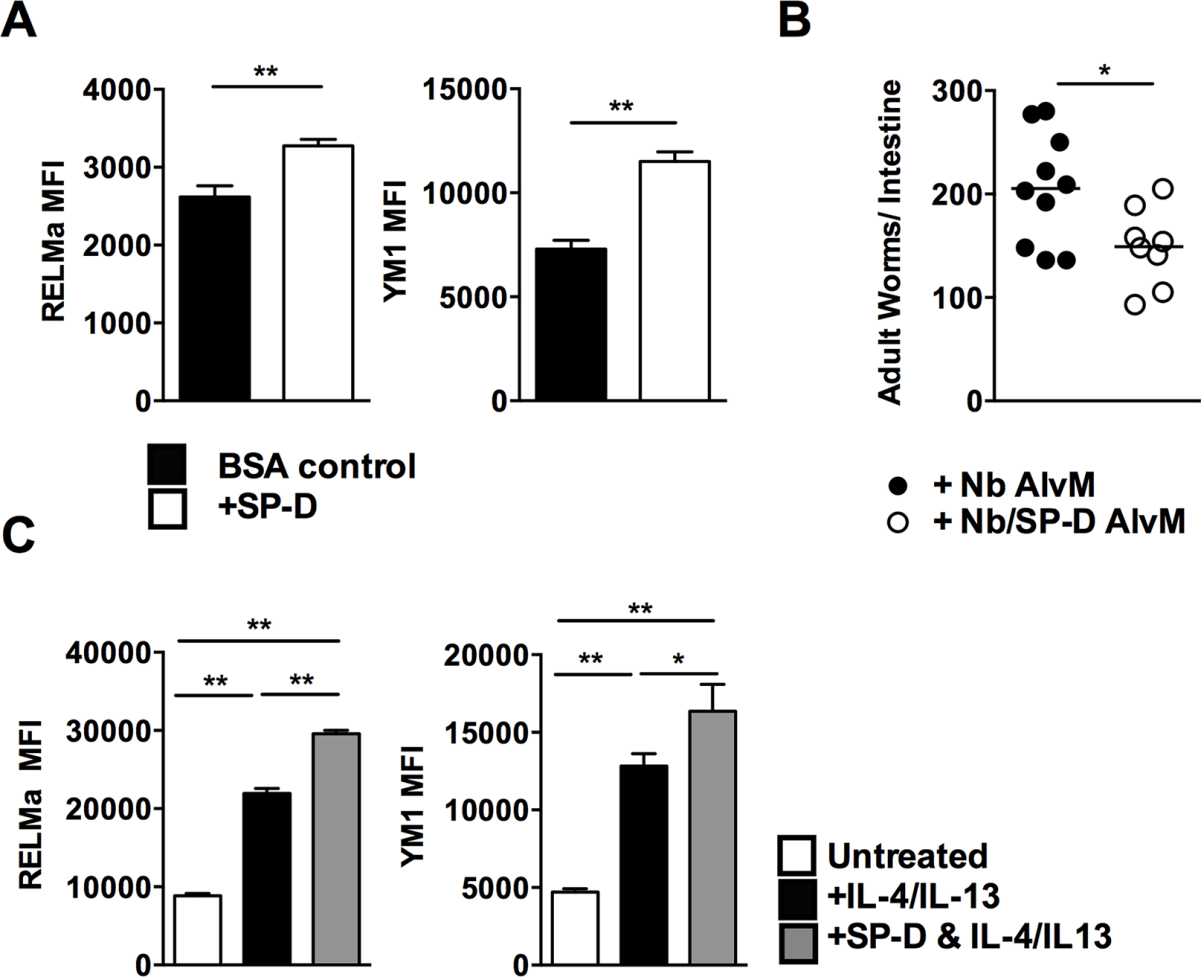
SP-D-induced protection is mediated by enhanced alternative activation of alveolar macrophages. Mean fluorescence intensity (MFI) of Ym1 and RELM-α expression on alveolar macrophages at day 5 PI rfhSP-D treated or untreated mice (**a**). Macrophages isolated from lungs of infected rfhSP-D treated or untreated mice were intra-nasally transferred into naïve BALB/c mice. Mice were then infected with *N*. *brasiliensis* and worm burdens were quantified at day 5 PI (**b**). 4 x 10^5^ macrophages sorted from naïve lungs were cultured with either IL-4/IL-13 (black), rfhSP-D and IL-4/IL-13 (grey) or untreated (white) and RELM-α and Ym1 expression was quantified by FACS (**c**). Data are representative of 2–3 experiments. N = 5–8 mice per group. *P<0.05, **P<0.01, ***P<0.001.

Intranasal administration of rfhSP-D resulted in an increased expression of the AAM markers YM1 and Relm-α in AlvMs when compared to BSA-treated controls at day 5 post-infection (**Fig 5A**). To test if these SP-D-dependent enhanced AAM response contributed to increased protection against *N*. *brasiliensis*, we isolated AlvMs from mice which had been infected with *N*. *brasiliensis* and treated with rfhSP-D or not, and transferred these cells intra-nasally into naïve mice. Recipients of rfhSP-D-treated macrophages had reduced intestinal worm burdens at day 5PI when compared to recipients of untreated macrophages (**Fig 5B** and **S5 Fig**).

It is typically considered that polarization to alternative activation by macrophages following an *N*. *brasiliensis* infection is a result of them responding to elevated levels of the cytokines IL-4 and IL-13 [30]. We directly tested *in vitro* whether rfhSP-D enhanced alternative activation of AlvMs isolated from naïve mice. Naïve AlvMs were polarized to AAM by *ex vivo* culture with recombinant IL-4/IL-13 in the presence or absence of rfhSP-D. Co-culture with rfhSP-D increased Ym1 and RELM-α expression when compared to macrophages treated with IL-4/IL-13 alone (**Fig 5C**). Collectively, these data show that SP-D can enhance AAM-dependent immunity to *N*. *brasiliensis*.

### SP-D enhances macrophage killing of *N*. *brasiliensis* by CRD mediated binding of L4 parasites

**Fig 6:**
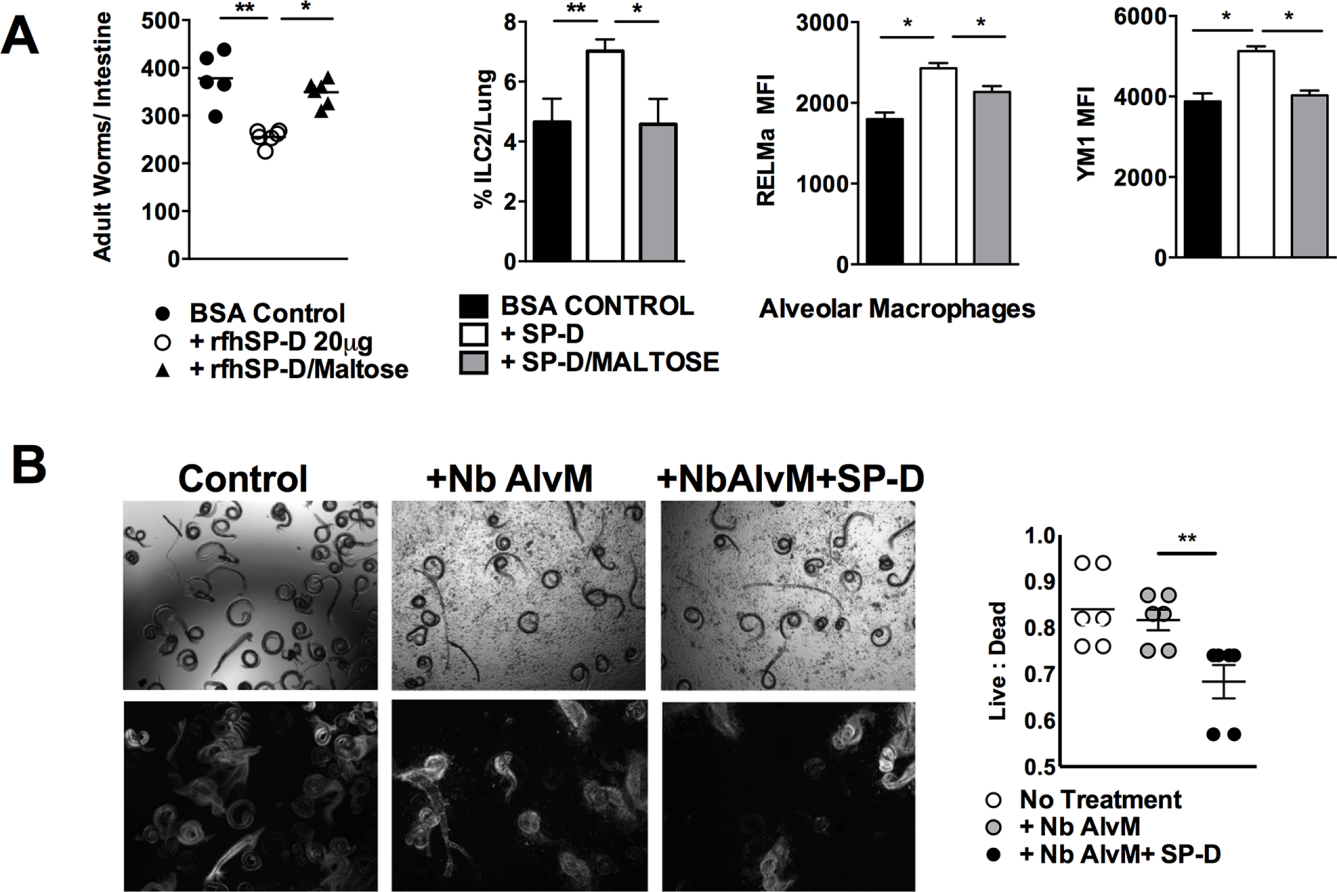
SP-D-mediated protection against *N*. *brasiliensis* infection requires carbohydrate recognition domain binding to opsonise L4 for enhanced macrophage killing. Mice were treated with BSA, rfhSP-D or maltose bound rfhSP-D. Intestinal worm burdens were quantified at day 5 PI, and alveolar cell populations were determined at day 5 PI (**a**). Untreated *N*. *brasiliensis* L4 or *N*. *brasiliensis* L4 pre-incubated with 20 μg/ml SP-D were cultured without or with alveolar macrophages (isolated from day 7 *N*. *brasiliensis* infected mice) for 48 hrs in serum free media and analysed by microscopy for viability (**b**). Top row shows bright field, bottom row shows standard deviation of overlay of 20 sequence pictures; white indicates movement. Data are representative of two individual experiments. N = 5 mice per group. *P<0.05, **P<0.01.

SP-D typically confers protection against infection by binding of the carbohydrate recognition domain (CRD) to the pathogen and, improving opsonisation and neutralization [31]. We tested if such an interaction contributed to SP-D-induced protection against *N*. *brasiliensis*. The ability of the rfhSP-D CRD domain to bind antigen can be blocked with maltose. We used this approach to block the ability of rfhSP-D to bind via the CRD to *N*. *brasilieinsis*. Mice treated with maltose-blocked rfhSP-D had higher worm burdens than mice treated with rfhSP-D alone; moreover, mice treated with rfhSP-D blocked with maltose did not demonstrate enhanced ILC2 and AAM responses seen in mice treated with rfhSP-D alone (**Fig 6A**).

We next tested if rfhSP-D opsonisation of L4 *N*. *brasiliensis* could enhance killing by alveolar macrophages. Macrophages isolated into serum-free medium from the lungs of *N*. *brasiliensis* infected mice were added to L4 *N*. *brasiliensis* in the presence or absence of rfhSP-D. In the presence of rfhSP-D and alveolar macrophages the ratio of live to dead parasites decreased when compared to parasites cultured with macrophages only **(Fig 6B)**.

## Discussion

To date it has never been demonstrated if SP-D or any other member of the collectin sub-family of C-type lectins can directly mediate innate protection against a parasitic helminth. In this study we show for the first time that SP-D is an important component of innate immunity to helminth infection.

We find that levels of SP-D in the lung and serum increase significantly and rapidly, in a manner analogous to an alarmin response, following infection with *N*. *brasiliensis*. This is in agreement with other reports where SP-D levels increase in response to acute lung stress following pulmonary challenges with, for example, lipopolysaccharide [32], bleomycin [33] and ovalbumin [34] Similarly, SP-D levels increase in the BAL following infections by *Aspergillus fumigatus* [35], *Actinobacillus pleurpneumoniae* or *Staphylococcus aureus* [36]. Our data represents the first known report of increased SP-D levels in response to a helminth infection.

Elevated SP-D production can also be driven by the type 2/TH2 cytokines IL-4 and IL-13, and in turn SP-D can impart negative feedback control of type 2/TH2 responses; indeed in the absence of SP-D TH-2 cytokine levels are raised [9]. Our findings expand on this understanding by demonstrating that production of SP-D following helminth infection is significantly dependent on key protective immune responses against *N*. *brasiliensis;* namely IL-4/IL-13 signaling via IL-4Rα. Previous demonstrations of increased type 2 immunity in SP-D-/- mice [9] may have suggested that immunity to *N*. *brasiliensis* infection [37] would have been enhanced in SP-D-/- mice. This was not the case and we account for this by exploring other roles for SP-D in controlling *N*. *brasiliensis* infections. Our results demonstrated that SP-D deficiency impaired innate type-2 responses associated with immunity to *N*. *brasiliensi*s infection [14]; moreover, we also found that elevated SP-D enhances these responses. This suggests that SP-D is an important modulator of protective ILC2 and alveolar macrophage responses against *N*. *brasiliensis*.

Opsonisation of pathogens and allergens by SP-D enhances host neutralization of them in the lung, primarily by improved recognition by host cells, such as alveolar macrophages [38]. It is well established that SP-D opsonization enhances innate immunity against a range of pathogens, such as bacteria, fungi and viruses [39]. We demonstrate that SP-D can also act as an interface between the L4 parasite and alveolar macrophages (which are key effector cells for controlling the parasite in the lung [27]). Moreover, direct interaction of SP-D with alveolar macrophages enhanced their polarization to an alternative phenotype. These findings demonstrate a pivotal role for alveolar macrophages in mediating the effects of SP-D via macrophage alternative activation dependent parasite immunity.

In accordance with studies demonstrating the requirement of the carbohydrate recognition domain (CRD) in mediating pathogen binding and enhanced phagocytosis and clearance by neutrophils and macrophages [31], our studies also demonstrate that SP-D-dependent clearance of *N*. *brasiliensis* is dependent on the CRD. Furthermore, SP-D binding through the CRD promotes innate type 2 responses including ILC2 induction and alternative activation of alveolar macrophages. We also show that SP-D can directly enhance L4 killing by alveolar macrophages following exposure to *N*. *brasiliensis* infection. These data clearly show that SP-D can act as an opsonin of L4 *N*. *brasiliensis* to enhance parasite killing by alveolar macrophages.

Our results therefore clearly demonstrate that binding of SP-D to the L4 lung stage of *N*. *brasiliensis* promotes parasite clearance via induction of innate type 2 responses including alternative activation of macrophages but also enhanced ILC2 expansion. To the best of our knowledge this is the first description of SP-D influencing ILC2 biology and we suggest that the decreased type 2 cytokine levels in the lungs may be a function of a loss of an interaction between ILC2 and SP-D in SP-D-/- mice.

Additionally, our *in vivo* studies show that SP-D deficiency increased the proportions of alveolar macrophages in the lung, key cells that maintain lung homeostasis and promote parasite clearance [10, 27]. Our observation of increased alveolar macrophage numbers is in agreement with other studies showing increased numbers of alveolar macrophage numbers in SP-D-/- mice [40]. However, reduced expression of the markers of alternative macrophage activation such as RELM-α within alveolar macrophages suggests that SP-D-deficiency does not favour induction of resolving macrophage populations in response to *N*. *brasiliensis* infection. Moreover, we show that in the absence of SP-D total levels of RELM-α in the lung were significantly reduced. Like SP-D, RELM-α regulates type 2 immunity; RELM-α^-/-^ mice develop heightened pathology following experimental *S*. *mansoni* infection [41]. Therefore, in addition to having impaired induction of AAM to act directly on the parasite SP-D-/- mice lack appropriate induction of other mediators of immune regulation which may have a wider impact on host control of infection induced pathology [15].

Induction of type 2 immunity to helminths is significantly dependent on epithelial cells (including ATII cells, the main cellular source of SP-D) secreting cytokines such as IL-33 [8] along with other immune modulators such as RELM-ß [42], trefoil factor 2 (TFF2) [43] and TSLP [44]. Our findings show that helminth induced SP-D is an additional major player in the host epithelial response to helminths. Balanced type 2 immunity is characteristic of effective host control of parasitic helminth infections and also reduced susceptibility to allergic disease [14]. Our findings therefore may have broader relevance to understanding innate immune control of diseases associated with poor control of type 2/Th2 immunity. Moreover, as helminth-induced SP-D is able to modify innate cell function and directly control lung inflammation, our studies set a precedent for placing SP-D in a central role of mediating parasite-associated protection from, for example, allergy and pulmonary viral infection

In conclusion, we show for the first time in both gain of function and loss function approaches that SP-D is required for immunity against *N*. *brasiliensis*. This enhanced immunity is coincident with an increased induction of cells associated with the resolution of infection; namely ILC2 and alternatively activated macrophages. Thus, helminth induction of SP-D is essential for host resolution of helminth infection.

## Materials and Methods

### Ethics statement

6-10-week-old BALB/c, C57/BL6, IL-4/13^-/-^[45], IL-4Rα^-/-^[46], SP-D-/- and CCSP-rtTA, (tetO)_7_-rSP-D,SP-D^−/−^ [21, 47] mice were obtained from colonies maintained by the University of Cape Town specific-pathogen-free animal facility. The authors are grateful to the laboratories of J Whitsett and S Hawgood for use of SP-D transgenic mice originally generated in their laboratories. Section 20 dispensation to carry out animal work at UCT was granted nationally by the South African Government Department of Agriculture Fisheries and Food and institutionally by the UCT Health Sciences Animal Ethics Committee (Project licence 012/054) to be in accordance with guidelines laid down by the South African Bureau of Standards. All researchers were accredited by the South African Veterinary Council. Dispensation to carry out animal research at Imperial College was approved by the Imperial College Animal Welfare and Ethical Review Body and granted by the UK government Home Office; as such all research here was carried under a specific project licence (PPL70/6957).

### *N*. *brasiliensis* infection

Mice were infected subcutaneously with 500 *N*. *brasiliensis* L3 larvae and killed at various times post infection as previously described [48]. Intra-nasal infection with rfhSP-D coated L4 *N*. *brasiliensis* was carried out using techniques adapted from Harvie et al [19]. Briefly, L4 *N*. *brasiliensis* were isolated 2 days post- *N*. *brasiliensis* infection from lung tissues. L4 *N*. *brasiliensis* were then incubated with rfhSP-D or bovine serum albumin (BSA) control for 1 hour at 37°C. L4 *N*. *brasiliensis* infection of mice was carried out by intranasal administration of 250 viable L4 worms in 50 μl to lightly anaesthetized mice. Adult worm burdens were determined by removing the small intestine and exposing the lumen by dissection. The intestines were incubated at 37°C for 4 hrs in 0.65% sodium chloride (NaCl) to allow the worms to migrate out, after which the numbers of worms were counted under a dissecting microscope.

### Intra-nasal administration of rfhSP-D

Mice were lightly anaesthetized and treated with 20 μg of rfhSP-D or BSA control (diluted in PBS) intra-nasally in a volume of 50 μl on day 0, 1, 2 and 3. For blocking of rfhSP-D CRD head region, 20 mM Maltose was incubated with rfhSP-D in the presence of 1 mM calcium chloride (CaCl_2_) for 1 hr at 37°C. Mice were killed on days indicated in results.

### BAL fluid, lung homogenates and serum

Mice underwent bronchoalveolar lavage (BAL) by administration of 1 ml sterile PBS containing 0.25 mM Ethylenediaminetetraacetic acid (EDTA) intra-tracheally. Lungs were lavaged three times. BAL fluid was centrifuged at 1200 rpm for 5 mins and the supernatant was frozen at -80°C.

The left lobe of the lung was snap frozen in liquid nitrogen and subsequently stored at -80°C until analysis. To prepare lung homogenates, 400 μl of lysis buffer [49] was added to lung tissue prior to mechanical homogenization. Homogenates were centrifuged at 14000 rpm for 20 mins and the protein concentrations of the supernatants determined using bicinchoninic acid (BCA) assay (Pierce, Rockford, IL).

Approximately 500 μl of blood was collected by cardiac puncture and the serum isolated by centrifugation (4000 rpm for 20 mins).

### Preparation of single cell-suspension of lung tissue

Whole lungs were removed from individual mice, finely cut and digested in Iscove’s modified Eagle medium (IMDM) (Invitrogen) containing 50 U/ml collagenase type I (Invitrogen) and 13 μg/ml DNase (Roche) at 37°C for 90 mins. Digested lung tissue were pushed through 70 or 100 μm nylon cell strainer (Becton Dickson, New Jersey) and subjected to red cell lysis.

### Flow cytometry

1 x 10^6^ single cell suspensions from individual lungs were stained in MACS buffer with anti-Siglec-F PE (E50/2440) and anti-CD11c APC (HL3) antibodies to stain for AlvMs and eosinophils. To stain for ILC2, the antibodies anti-Lineage PE (CD3, CD19, CD11b, FceR1, Ter119, CD4, CD8, B220, Ly6G/6C), anti-CD45 AF700, anti-CD90 PacBlu, anti-CD127 PE-Cy7 or APC (SB/199), anti-ICOS Biotin or FITC (7E.17G9) anti- Sca-1 V450 (D7) and anti-T1/ST2 FITC PerCP-Cy5.5 (DJ8) were used. Anti-FcR (2.4G2) was used to block non-specific binding of immunoglobulins to the FCγII/III receptors.

For intracellular staining, cells were stained with surface markers, fixed in 2% paraformaldehyde before being permeabilized with buffer containing saponin. Cells were subsequently stained with anti-Ym1 biotin (ECF-L) and anti-RELM-α (E19). For intracellular staining of ILC2 cells were fixed and permeabilized with Fix/perm (eBioscience) and stained with IL-13 PE-Cy7. Cells were acquired using a FORTESSA Flow cytometer (BD Biosciences) and data analyzed using FlowJo software (Tree star, inc., Ashland, Oregon, USA). Antibodies were purchased from BD Pharmingen, eBioscience and Biolegend.

### Enzyme-linked immunosorbent assay (ELISA) analysis

Whole lung and intestine homogenates were used to quantify cytokines, and BAL fluid or serum from *N*. *brasiliensi*s infected mice were analyzed for SP-D content by ELISA. 96-well flat-bottom plates (Nunc Maxisorp; Thermo Fisher Scientifica, Roskilde, Denmark) were coated overnight at 4°C with 50 μl of appropriate coating antibody diluted in 1X PBS. Plates were washed four times in Tris-Buffered Saline containing % Tween (TBST), blocked with 200 μl blocking buffer overnight at 4°C. Following this, appropriate dilutions of samples and standards were prepared, loaded into wells and incubated overnight at 4°C. The plates were further washed and 50 μl of appropriate biotinylated secondary antibodies were added and incubated at 37°C for 3 hrs. 50 μl of Streptavidin-coupled horseradish peroxidase (HRP) was then added after washing and plates were incubated for 1 hr at 37°C. The plates were developed with 3,3’,5,5’-Tetramethylbenzidine (TMB) microwell peroxidase substrate system, and the reaction was stopped with 1 M H_3_PO_4_. The plates were read at an absorbance of 450 nm using a VersaMax microplate reader (Molecular Devices Corporation, Sunnyvale, CA, U.S.A). All antibodies were from BD Pharmingen, San Diego, CA.

### Isolation & adoptive transfer experiments

Mice were treated with 20 μg of rfhSP-D or BSA at D0, 1, 2, 3, 6 and 7 post-infection. Single-cell suspensions of pooled lungs were prepared at day 8 post-infection and AlvMs were stained with anti-CD11c APC-conjugated and anti-Siglec-F PE conjugated monoclonal antibody (MAb) (BD Pharmingen) before they were isolated (> 95% purity) as CD11c^+^Siglec F^+^Autoflourescent^high^ using a FACS Aria cell sorter (Becton Dickinson), purity was also confirmed by microscopic analysis (**S5A Fig**). 1 x 10^5^ macrophages were then transferred intra-nasally into naïve BALB/c mice 24 hrs prior to *N*. *brasiliensis* infection.

### In vitro culture of macrophages with SP-D

Naïve AlvMs (CD11C^+^Siglec-F^+^AutoFlourescent^high^) were isolated from single cell suspensions of lung tissue by FACS Aria as described above and plated in duplicates at 4x10^5^ cells per well. Cells were stimulated with either recombinant mouse IL-4/IL-13, IL-4/IL-13 + 20 μg/ml of rfhSPD or left untreated. The cultures were incubated for 60 hrs at 37°C. Thereafter, cells were washed and stained for alternative activation markers, YM1 and Relm- α as described above, and acquired with LSRFORTESSA (BD Biosciences).

### In vitro culture of L4, SP-D and macrophages

Mice were infected with 500 L3 *N*. *brasiliensis* and lungs isolated at day 7 post-infection. Single cell suspensions were stained for Siglec-F and CD11c and live cells isolated into serum free media using a FACS Aria, as described above. L4 were isolated from lungs as described previously. Experiments were carried using serum free media. L4 were either left untreated or incubated with 20 μg/ml SP-D for 1 hr before addition to 4x10^5^ macrophages. After 48 hrs larvae were washed, counted and analysed for movement by bright field microscopy and a sequence of 20 images/min were taken. These were then analysed by SD overlay using Fiji software. Live/dead ratios were calculated using total, moving and dead numbers.

### Confocal microscopy

L3, L4 and adult stage larvae of *N*. *brasiliensis* were fixed overnight in 2% paraformaldehyde at 4°C. The larvae were extensively washed using 1X PBS containing 0.2% BSA and 1 mM CaCl_2_. Non-specific binding was blocked by incubation of the larvae in 0.2% BSA in PBS for 1 hr at room temperature. Thereafter, the larvae were incubated with 20 μg/ml rfhSP-D in PBS containing 0.2% BSA and 1mM CaCl_2_ for 1 hr at 32°C. After extensive washing, the larvae were incubated with biotinylated rabbit anti-rfhSP-D antibody (HYB 246.04, Antibody shop) used at 1/200 and left overnight at 4°C. To detect the SP-D binding, the organisms were subsequently incubated with Streptavidin cy3 (1/500) for 2 hrs at room temperature (RT). All sections were viewed with Axiovert LSM 510 Meta NLO microscope (Zeiss).

### Statistics

Data were expressed as mean ± standard deviation and analyzed using Mann-Whitney nonparametric T test or ANOVA with a 95% confidence interval. p values are represented as p< 0.05 (*), p<0.01 (**) and p<0.005 (***).

## Supporting Information

S1 Fig SP-D is induced in the serum following *N*. *brasiliensis* infection and correlates with the levels in BAL fluid. Kinetics of SP-D levels was measured by ELISA in serum following *N*. *brasiliensis* infection (**a**). SP-D levels of IL-4/IL-13^-/-^ mice (**b**) were measured in serum at day 5 PI and compared to wild type controls. Data are representative of one individual experiment. N = 5 mice per group. **P<0.01.
(TIFF)

S2 Fig Expression of SP-D in the lung contributes significantly to reduced *N*. *brasiliensis* burden in the host. Relative concentration of RELM-α and YM1 in individual lung samples of day 9 PI WT and SP-D-/- mice as detected by Western Blot and quantified by densitometry (**a**). Data are representative of 2 individual experiments. WT, CCSP-rtTA, (tetO)_7_-rSP-D,SP-D ^−/−^ mice–doxycycline (SP-Doff) and CCSP-rtTA, (tetO)_7_-rSP-D,SP-D ^−/−^ mice + doxycycline (SP-Don) were infected with 500 x L3 *N*. *brasiliensis* and intestinal worm burdens established at day 5 PI (**b**). Data are representative of 2 individual experiments. N = 4–6 mice per group.
(TIFF)

S3 Fig Selective SP-D binding to L4 *Nippostrongylus brasiliensis* enhances host protective immunity. Labeling of *N*. *brasiliensis* parasites with anti-IgG2b isotype control (**a**). Untreated *N*. *brasiliensis* L4 or *N*. *brasiliensis* L4 pre-incubated for 1 hr with either 20 μg/ml BSA or 20 μg/ml SP-D. Worm motility was assessed by time lapse photography (**b**). Top row shows bright field, bottom row shows standard deviation of overlay of 20 sequence pictures; white indicates movement. Data are representative of two individual experiments.
(TIFF)

S4 Fig Effect of intra-nasal administration of SP-D on naïve lung. BALB/c mice were treated with 20 μg rfhSP-D for 4 days. IL-4, IL-13 and IL-33 cytokine levels in lung homogenates were detected by ELISA at day 5 PI (**a**). Total numbers and proportions of lung ILC2s (**b**). Data are representative of 2 individual experiments. N = 4 mice per group.
(TIFF)

S5 Fig Morphology and immune characterization of adoptively transferred alveolar macrophage populations. Light microscope analysis of macrophage morphology in cells isolated from *N*. *brasiliensis* infected and *N*.*brasiliensis* + SP-D treated mice (**a**). Expression levels of YM1 and RELM-α on alveolar macrophages isolated from *N*. *brasiliensis* infected and *N*. *brasiliensis* infected + SP-D treated mice (**b**). Data are representative of 2 individual experiments.
(TIFF)
