# Supplementary figures and images for: Surfactant Protein-D Is Essential for Immunity to Helminth Infection
Source: PLoS Pathog. 2016 Feb 22;12(2):e1005461. doi: 10.1371/journal.ppat.1005461 (PMC4763345; doi:10.1371/journal.ppat.1005461)

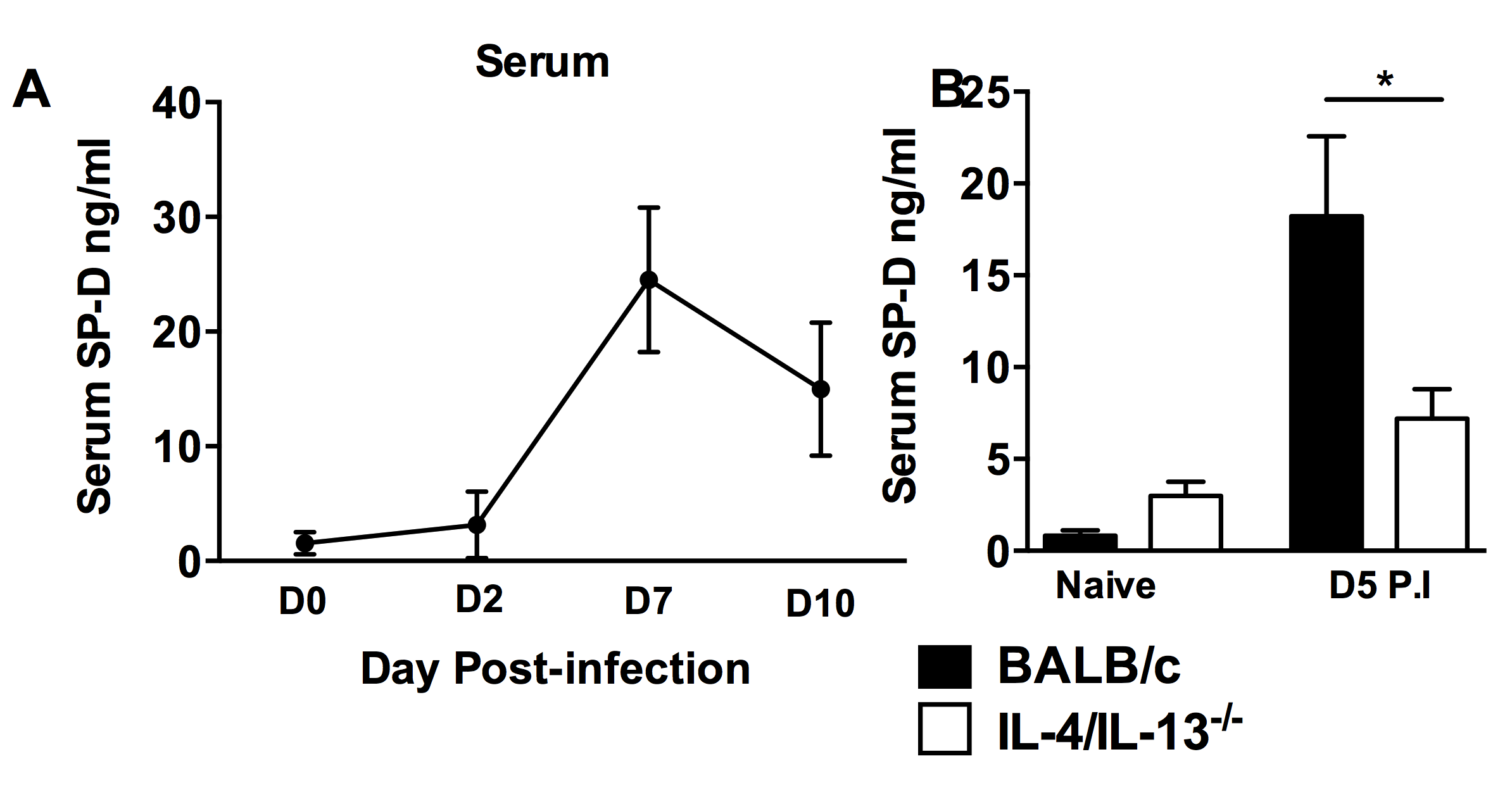

Supplement: S1 Fig — Kinetics of SP-D levels was measured by ELISA in serum following N. brasiliensis infection (a). SP-D levels of IL-4/IL-13-/- mice (b) were measured in serum at day 5 PI and compared to wild type controls. Data are representative of one individual experiment. N = 5 mice per group. **P<0.01. (TIFF) [file ppat.1005461.s001.tiff]

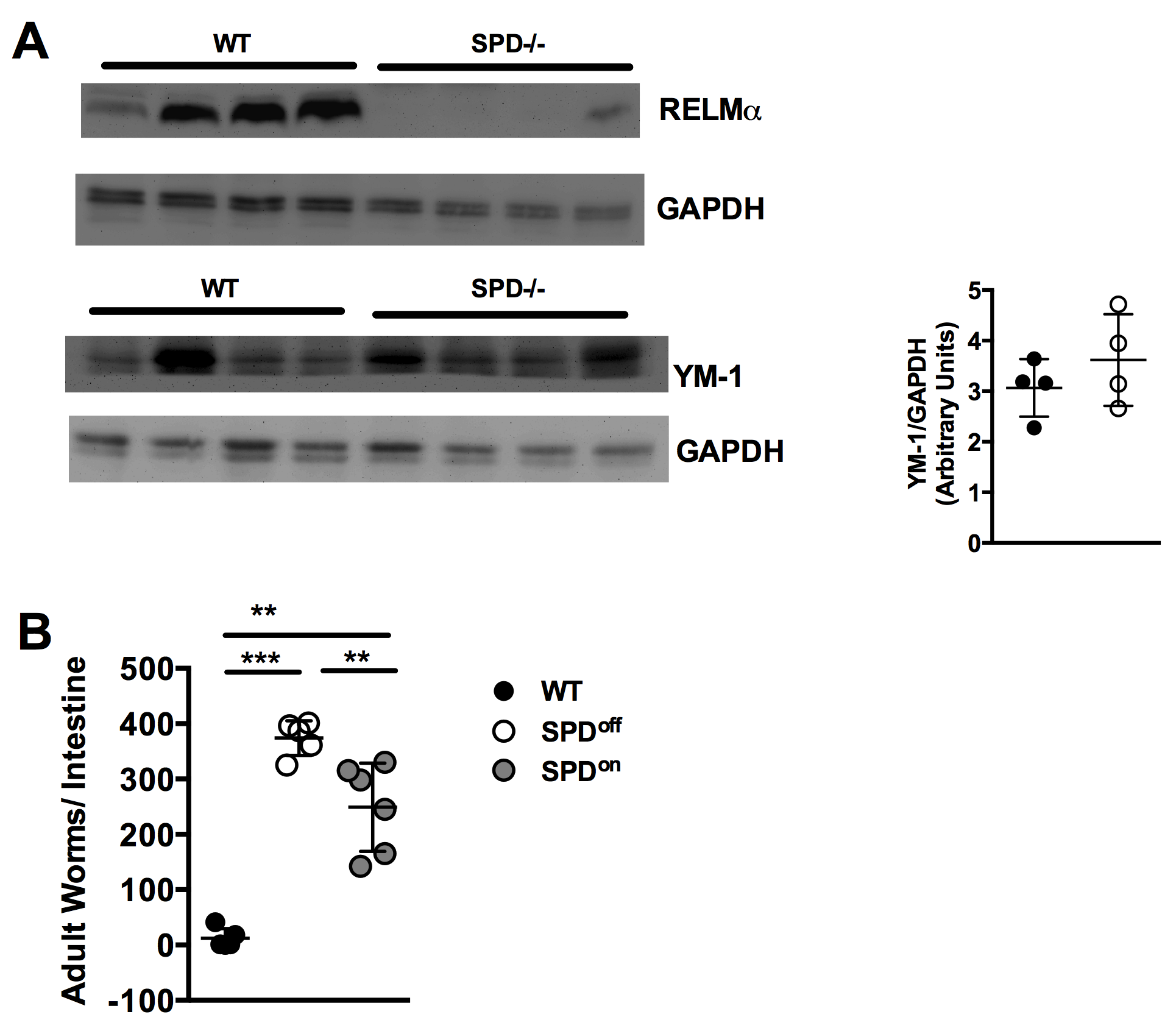

Supplement: S2 Fig — Relative concentration of RELM-α and YM1 in individual lung samples of day 9 PI WT and SP-D-/- mice as detected by Western Blot and quantified by densitometry (a). Data are representative of 2 individual experiments. WT, CCSP-rtTA, (tetO)7-rSP-D,SP-D −/− mice–doxycycline (SP-Doff) and CCSP-rtTA, (tetO)7-rSP-D,SP-D −/− mice + doxycycline (SP-Don) were infected with 500 x L3 N. brasiliensis and intestinal worm burdens established at day 5 PI (b). Data are representative of 2 individual experiments. N = 4–6 mice per group. (TIFF) [file ppat.1005461.s002.tiff]

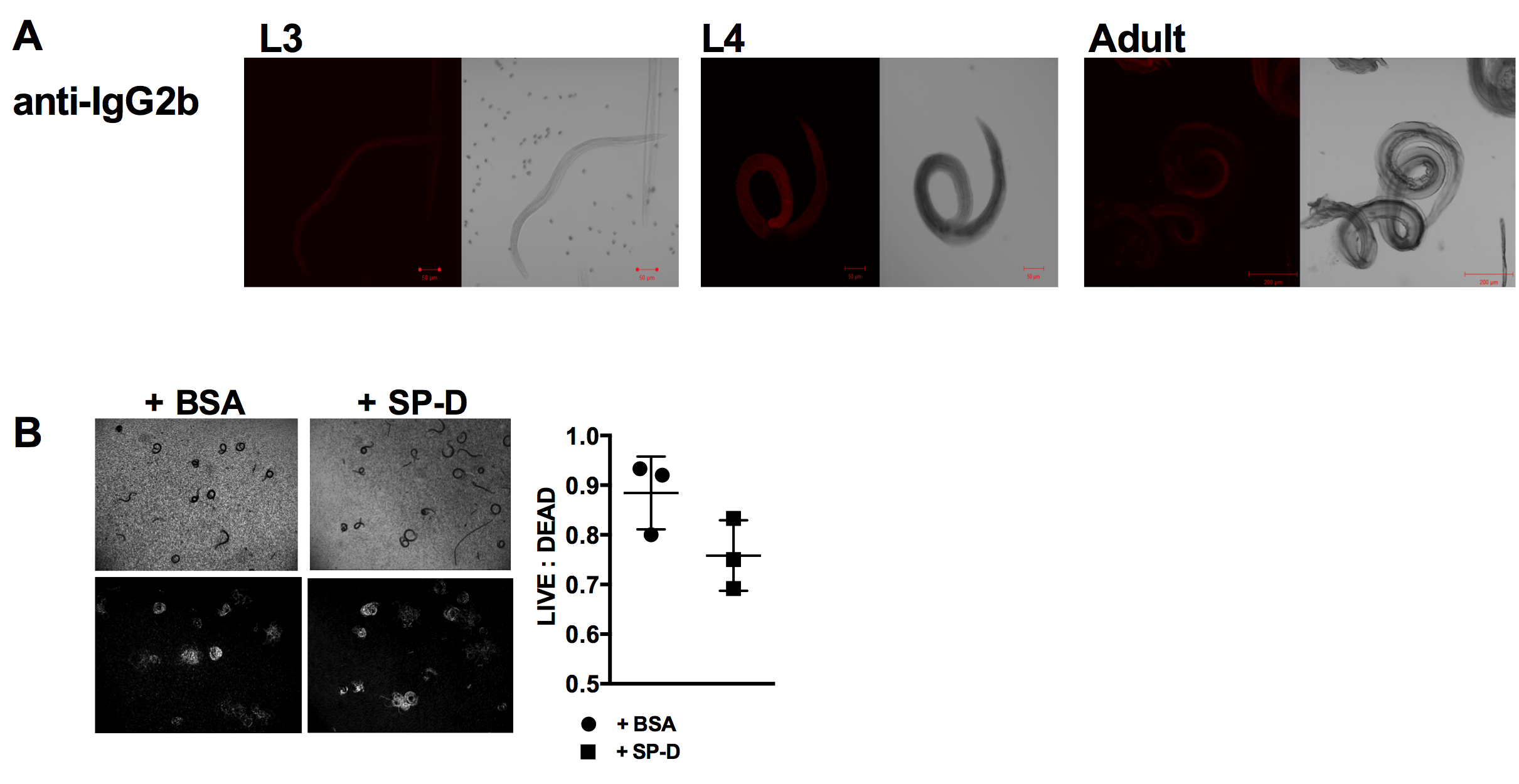

Supplement: S3 Fig — Labeling of N. brasiliensis parasites with anti-IgG2b isotype control (a). Untreated N. brasiliensis L4 or N. brasiliensis L4 pre-incubated for 1 hr with either 20 μg/ml BSA or 20 μg/ml SP-D. Worm motility was assessed by time lapse photography (b). Top row shows bright field, bottom row shows standard deviation of overlay of 20 sequence pictures; white indicates movement. Data are representative of two individual experiments. (TIFF) [file ppat.1005461.s003.tiff]

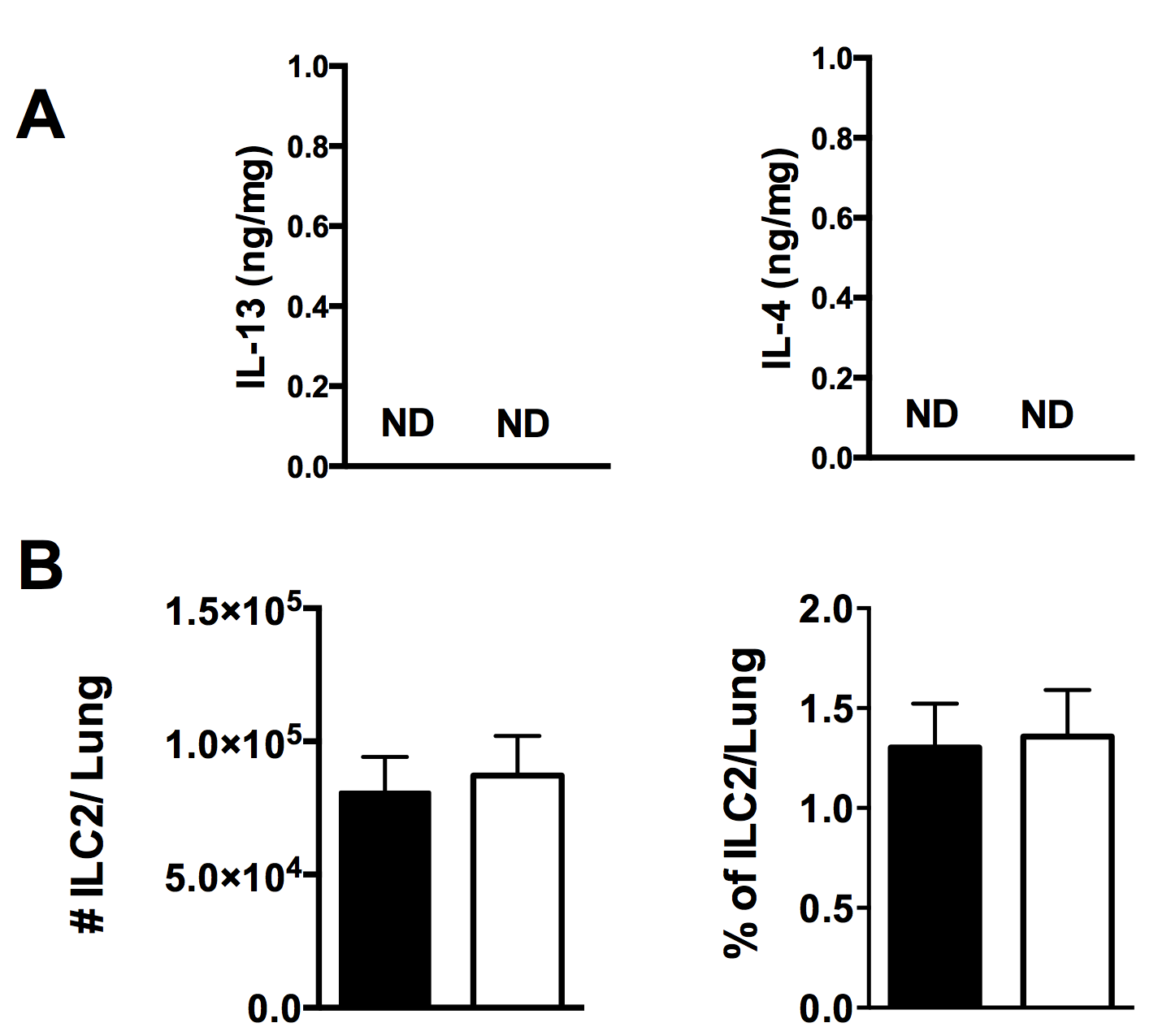

Supplement: S4 Fig — BALB/c mice were treated with 20 μg rfhSP-D for 4 days. IL-4, IL-13 and IL-33 cytokine levels in lung homogenates were detected by ELISA at day 5 PI (a). Total numbers and proportions of lung ILC2s (b). Data are representative of 2 individual experiments. N = 4 mice per group. (TIFF) [file ppat.1005461.s004.tiff]

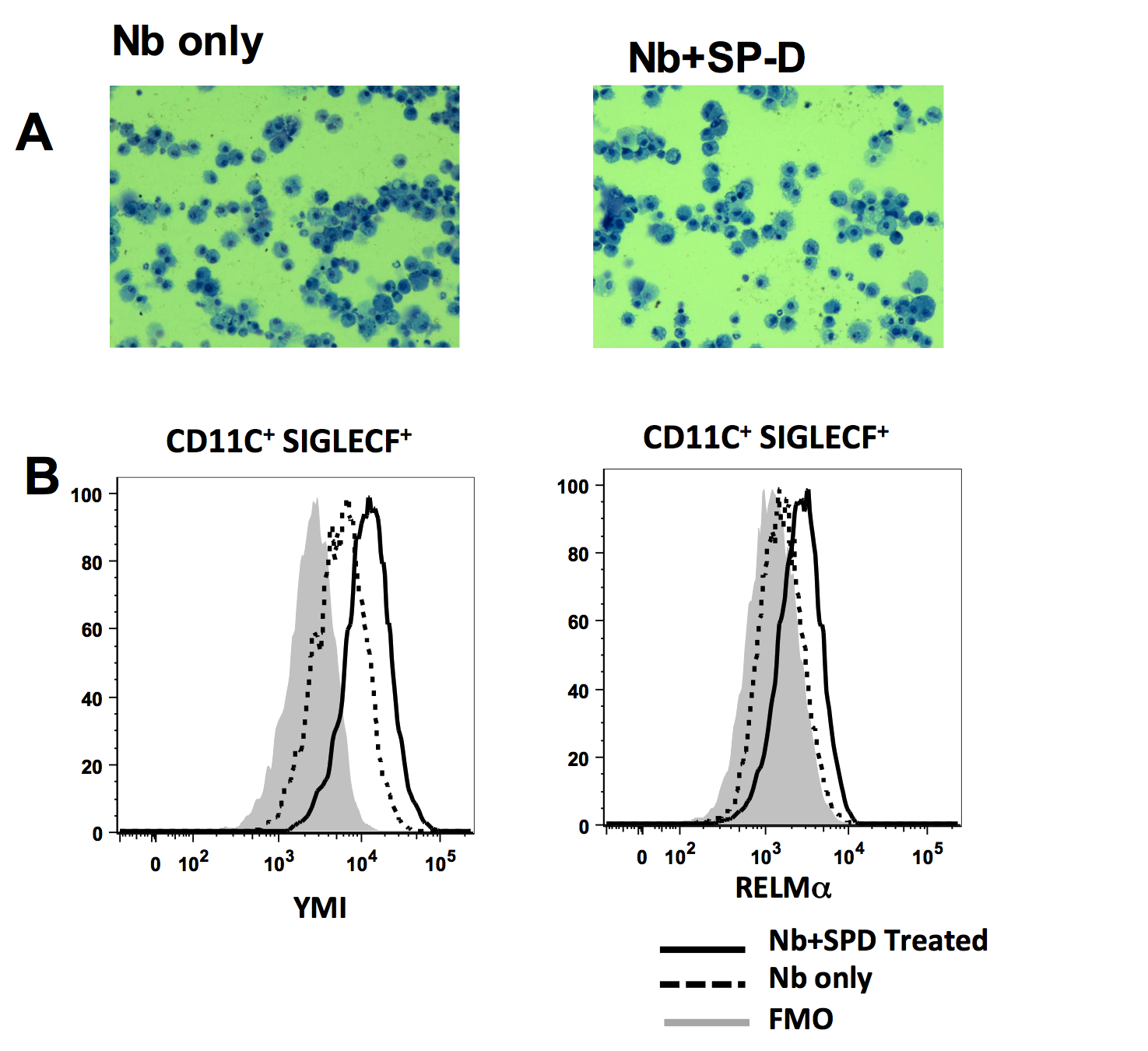

Supplement: S5 Fig — Light microscope analysis of macrophage morphology in cells isolated from N. brasiliensis infected and N.brasiliensis + SP-D treated mice (a). Expression levels of YM1 and RELM-α on alveolar macrophages isolated from N. brasiliensis infected and N. brasiliensis infected + SP-D treated mice (b). Data are representative of 2 individual experiments. (TIFF) [file ppat.1005461.s005.tiff]
